# Predicting Individual Treatment Response to rTMS for Motor Recovery After Stroke: A Review and the CanStim Perspective

**DOI:** 10.3389/fresc.2022.795335

**Published:** 2022-02-10

**Authors:** Franziska E. Hildesheim, Alexander N. Silver, Adan-Ulises Dominguez-Vargas, Justin W. Andrushko, Jodi D. Edwards, Numa Dancause, Alexander Thiel

**Affiliations:** ^1^Lady Davis Institute for Medical Research, Jewish General Hospital, Montréal, QC, Canada; ^2^Canadian Platform for Trials in Non-Invasive Brain Stimulation (CanStim), Montréal, QC, Canada; ^3^Department of Neurology and Neurosurgery, McGill University, Montréal, QC, Canada; ^4^Centre interdisciplinaire de recherche sur le cerveau et l'apprentissage (CIRCA), Université de Montréal, Montréal, QC, Canada; ^5^Département de Neurosciences, Faculté de Médecine, Université de Montréal, Montréal, QC, Canada; ^6^Department of Physical Therapy, Faculty of Medicine, University of British Columbia, Vancouver, BC, Canada; ^7^University of Ottawa Heart Institute, Ottawa, ON, Canada; ^8^School of Epidemiology and Public Health, University of Ottawa Heart Institute, Ottawa, ON, Canada

**Keywords:** repetitive transcranial magnetic stimulation, stroke, motor recovery, rehabilitation, prediction, review

## Abstract

**Background:**

Rehabilitation is critical for reducing stroke-related disability and improving quality-of-life post-stroke. Repetitive transcranial magnetic stimulation (rTMS), a non-invasive neuromodulation technique used as stand-alone or adjunct treatment to physiotherapy, may be of benefit for motor recovery in subgroups of stroke patients. The Canadian Platform for Trials in Non-Invasive Brain Stimulation (CanStim) seeks to advance the use of these techniques to improve post-stroke recovery through clinical trials and pre-clinical studies using standardized research protocols. Here, we review existing clinical trials for demographic, clinical, and neurobiological factors which may predict treatment response to identify knowledge gaps which need to be addressed before implementing these parameters for patient stratification in clinical trial protocols.

**Objective:**

To provide a review of clinical rTMS trials of stroke recovery identifying factors associated with rTMS response in stroke patients with motor deficits and develop research perspectives for pre-clinical and clinical studies.

**Methods:**

A literature search was performed in PubMed, using the Boolean search terms *stroke* AND *repetitive transcranial magnetic stimulation* OR *rTMS* AND *motor* for studies investigating the use of rTMS for motor recovery in stroke patients at any recovery phase. A total of 1,676 articles were screened by two blinded raters, with 26 papers identified for inclusion in this review.

**Results:**

Multiple possible factors associated with rTMS response were identified, including stroke location, cortical thickness, brain-derived neurotrophic factor (*BDNF*) genotype, initial stroke severity, and several imaging and clinical factors associated with a relatively preserved functional motor network of the ipsilesional hemisphere. Age, sex, and time post-stroke were generally not related to rTMS response. Factors associated with greater response were identified in studies of both excitatory ipsilesional and inhibitory contralesional rTMS. Heterogeneous study designs and contradictory data exemplify the need for greater protocol standardization and high-quality controlled trials.

**Conclusion:**

Clinical, brain structural and neurobiological factors have been identified as potential predictors for rTMS response in stroke patients with motor impairment. These factors can inform the design of future clinical trials, before being considered for optimization of individual rehabilitation therapy for stroke patients. Pre-clinical models for stroke recovery, specifically developed in a clinical context, may accelerate this process.

## Introduction

Repetitive transcranial magnetic stimulation (rTMS) is a non-invasive neuromodulation technique with the potential to modify cortical excitability in localized brain regions directly under the stimulation coil, as well as in distal brain regions connected to the stimulation site (1). Brief electrical currents are induced through strong magnetic fields (1, 2). By varying the number, frequency and intensity of magnetic pulses, different effects can be induced in the brain. Generally, low-frequency pulse rates of ≤ 1 Hz have inhibitory effects on underlying brain tissue by reducing the excitability of neurons, whereas high-frequency pulse rates ≥5 Hz have excitatory effects [see Ridding and Rothwell (3) for a more detailed review]. Another rTMS protocol, theta-burst stimulation (TBS), uses multiple short bursts of 50 Hz pulses (4). Depending on whether these pulse trains are applied intermittently (iTBS) or continuously (cTBS), TBS can act as either excitatory or inhibitory stimulus. rTMS is considered safe, with the only common adverse effect being minor local reactions, such as headache or scalp discomfort. The most serious adverse effect reported in literature is induction of generalized seizures. The risk is however considered very low, even among those taking drugs acting on the central nervous system (5). Updated guidelines for the therapeutic use of rTMS to maximize patient safety and minimize the risk of severe adverse events have recently been published (5).

**Figure 1:**
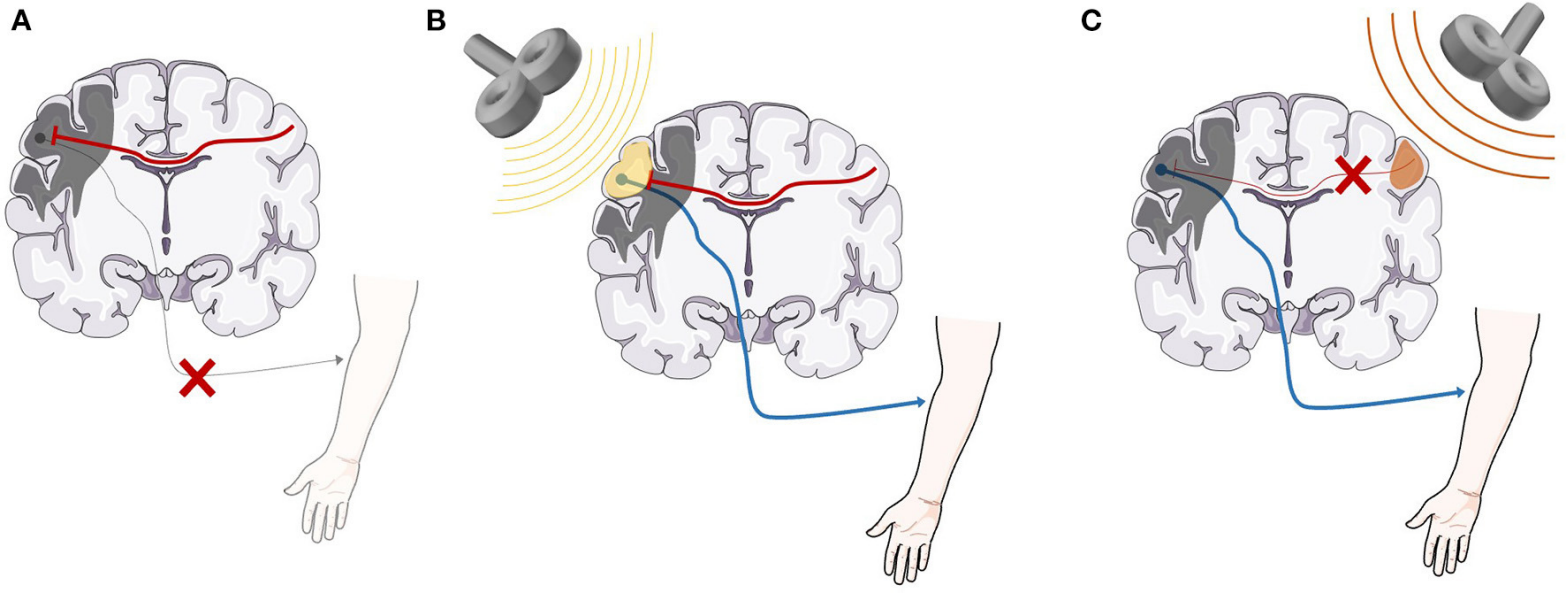
A representation of the basic neurobiological model underlying rTMS as an adjunct treatment for stroke recovery. **(A)** After stroke, direct damage to the primary motor cortex as well as inhibitory signaling from the contralesional motor cortex are both likely involved in lack of functional recovery (8–11). **(B)** High-frequency (>5 Hz) rTMS applied over the ipsilesional hemisphere strengthens the descending motor pathway, facilitating motor recovery. **(C)** Low-frequency (<1 Hz) rTMS applied over the contralesional hemisphere reduces inhibitory signals from the contralesional motor cortex, promoting beneficial cortical reorganization and motor recovery (3). rTMS, repetitive transcranial magnetic stimulation. Anatomical images adapted from smart.servier.com.

rTMS has been claimed to have benefits in a wide variety of psychiatric and neurological conditions (3), however, major unipolar depression and obsessive-compulsive disorder are currently the only indications with FDA approval (6). The use of rTMS as an adjunct to physical therapy for recovery of motor function in stroke has received particular attention, due to the high prevalence of stroke and residual disability of function, even with current standard of care rehabilitation treatment (7). Two general types of rTMS protocols are used in stroke rehabilitation (Figure 1). In the first approach, excitatory high-frequency rTMS stimulation is applied over the ipsilesional primary motor cortex (M1) or adjacent brain areas. The mechanism by which this promotes motor recovery over time is not fully understood, but may involve strengthening of synaptic connections in descending motor pathways (3). In the second approach, inhibitory low-frequency rTMS is applied over contralesional M1, which may reduce interhemispheric inhibition from the contralesional M1 onto the ipsilesional M1, and thereby promote cortical reorganization in the ipsilesional hemisphere.

Both excitatory and inhibitory rTMS protocols have been shown to improve motor recovery in post-stroke patients in the acute, subacute and chronic phase of recovery (12–15). However, inter-individual variability in response to rTMS treatment remains high, and evidence regarding factors that may contribute to this variability is fragmentary. Identifying factors causing this variability is thus key to improve the identification of patients most likely to benefit from rTMS treatment and to recruit more homogeneous populations into clinical trials. The purpose of this review is to identify potential predictive factors from the literature which could be subject to future targeted validation studies to inform implementation into clinical trial protocols.

## Methods

A literature search was performed in PubMed for the identification of articles published prior to July 2021. The database was searched using the Boolean search terms *stroke* AND *repetitive transcranial magnetic stimulation* OR *rTMS* AND *motor*. Only full-text articles were considered for inclusion. Studies were included based on the following inclusion criteria: (1) diagnosis of ischemic or hemorrhagic stroke in human subjects, (2) patients are reported to suffer from upper or lower extremity deficits, (3) study assesses and reports upper or lower limb motor function or associated electrophysiological parameters before and after rTMS intervention, and (4) study reports statistical analysis results (e.g., ANOVA, multivariate regression model, etc.) of patient factors associated with differential rTMS response. Both studies including rTMS as a stand-alone treatment and those combining rTMS with physiotherapy or occupational therapy programs were included. Studies with patients of all age, sex and education level, as well as patients in all phases post-stroke (acute, subacute, chronic), were considered for study inclusion. Exclusion criteria included non-therapeutic use of TMS and use of another invasive or non-invasive neuromodulation technique (e.g., transcranial direct current stimulation [tDCS]). Review articles, meta-analyses, editorials, and guidelines, as well as articles not available in English, French, or German were also excluded.

A total of 1,676 articles were found with this initial search protocol. For article organization, the open-access review software Rayyan was used (www.rayyan.ai). After removal of duplicates, the titles and abstracts of remaining articles were screened by two independent blinded raters (F.E.H. and J.W.A.), to determine their relevance for the research question of this review. After article screening, the results were unblinded. Mismatched papers were reviewed by a third independent rater (A.N.S.) and disagreements were resolved through consensus (F.E.H., J.W.A. and A.N.S).

## Results

**Figure 2:**
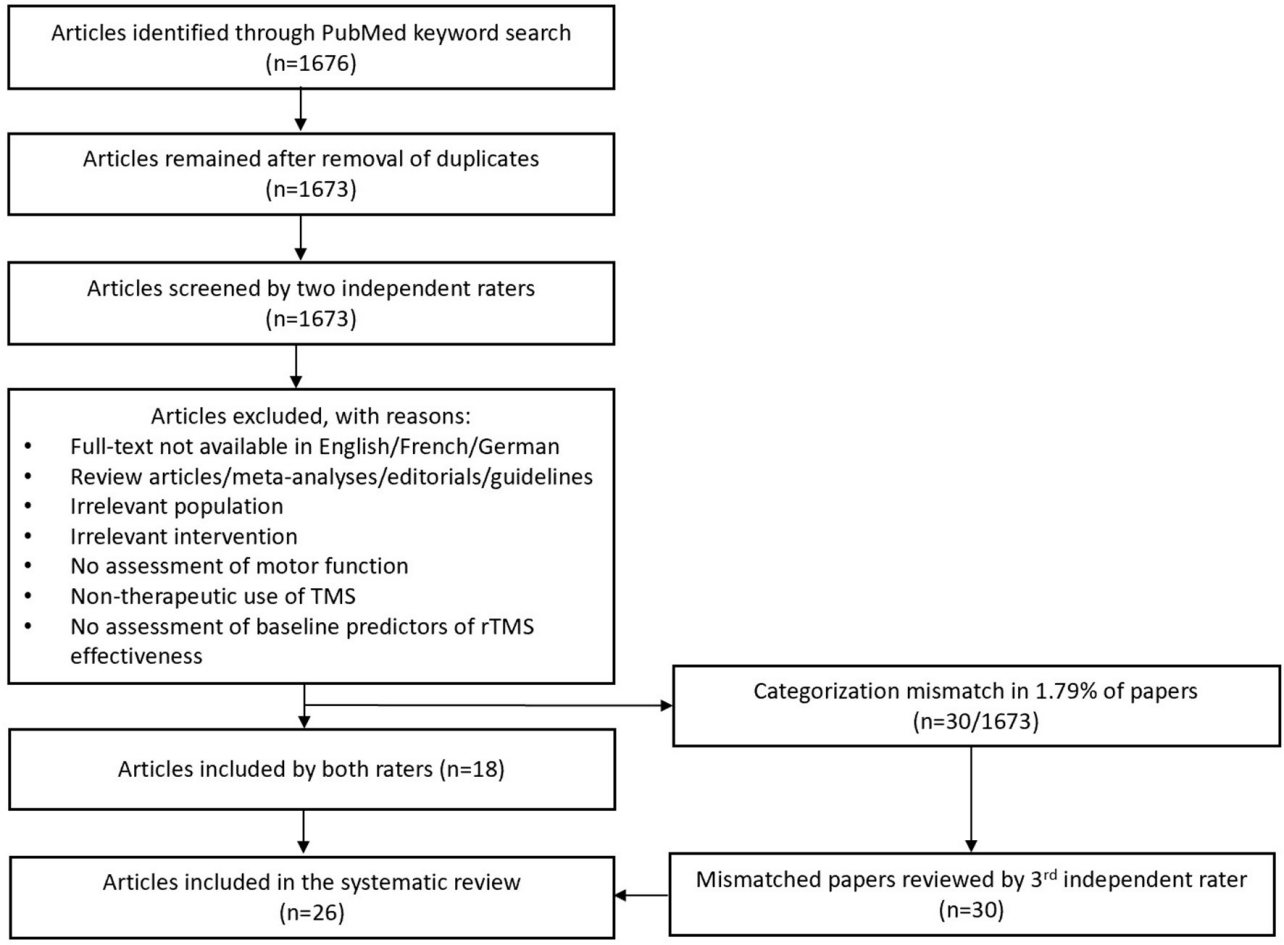
Study selection procedure. rTMS, repetitive transcranial magnetic stimulation.

A total of 1,676 articles were identified, and with duplicates removed, 1,673 articles remained to be screened for inclusion by the two raters. The agreement between reviewers on study inclusion was 98.2%, with a categorization mismatch in 30/1,673 articles (1.79%) and 18/1,673 (1.07%) articles identified for inclusion by both raters before unblinding. After review of the mismatched papers by the third rater, 26 articles with a total of 3,975 participants were identified as relevant and included in this review (Figure 2).

**Figure 3:**
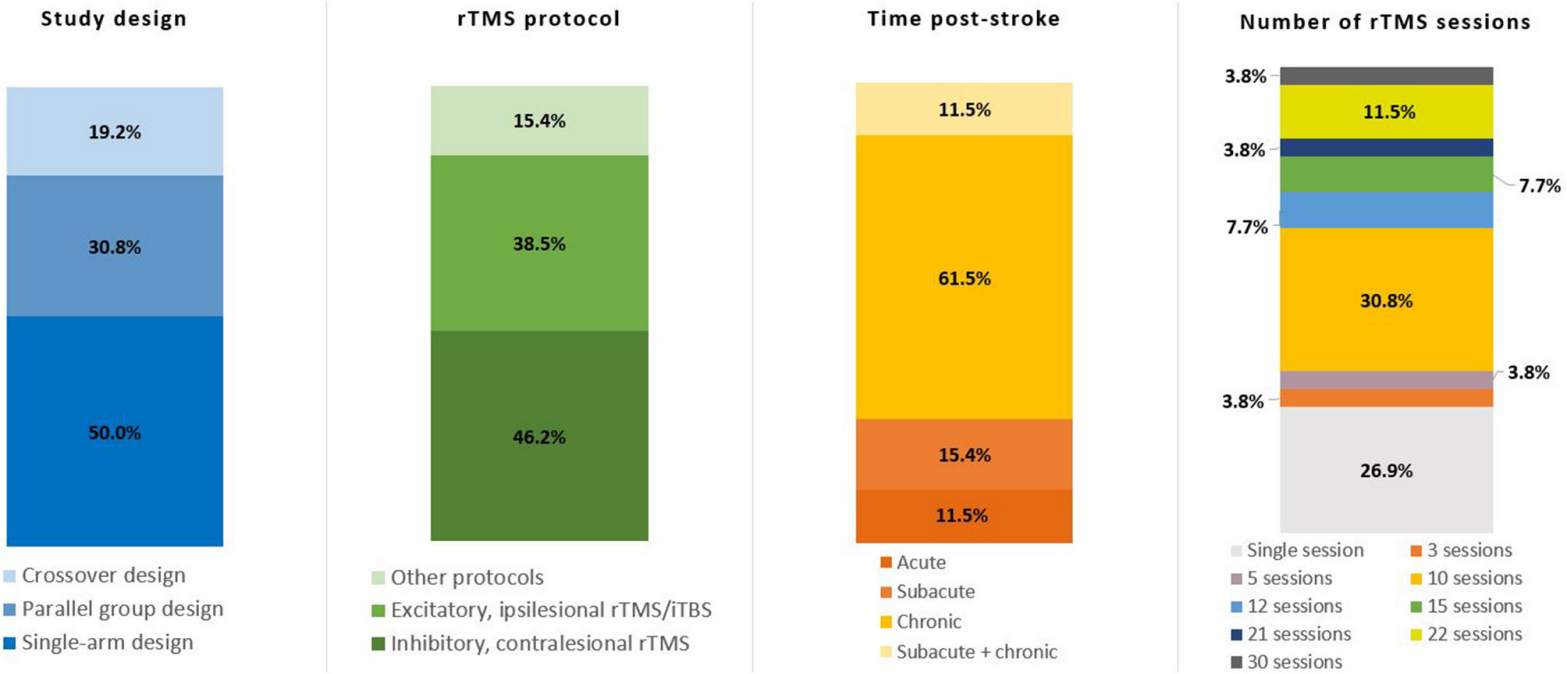
Key details of included studies. Total number of studies *n* = 26. rTMS, repetitive transcranial magnetic stimulation; iTBS, intermittent theta burst stimulation.

Table 1 provides an overview of included studies, and Figure 3 illustrates the variation in study design, rTMS protocol, time post-stroke and number of sessions across studies. Half of the included studies (13/26), encompassing a large majority of the patient population, were single-arm, non-randomized retrospective or prospective studies (21–32, 36). Eleven studies included a sham-control condition either in a crossover or parallel-group design (16, 20, 33–35, 37–42). Of the 11 sham-controlled trials, only four were randomized, double-blind trials (19, 20, 35, 38).

**Table 1:** Overview of studies included in this review.

**References**	***n***	**Phase post-stroke**	**rTMS protocol**	**Study design**	**Primary outcome measures**	**Factors associated with rTMS response**
Hensel et al. (16)	13	Acute	Excitatory 10 Hz, contralesional dPMC, M1 & aIPS; single session	Crossover (sham vs. rTMS), single-blind, randomized	Index finger tapping	Connectivity between frontal motor regions and aIPS
Chang et al. (17)	44	Acute	Excitatory 10 Hz, ipsilesional M1, 10 sessions	Parallel-group (Val/Val vs. Met allele), double-blind	FMA, BBT	Val/Val *BDNF* genotype
Di Lazzaro et al. (18)	20	Acute	iTBS, ipsilesional M1, single session	Parallel-group (Val/Val vs. Met allele), double-blind	Changes in cortical excitability (RMT, MEP, AMT)	Val/Val *BDNF* genotype
Kim et al. (19)	73	Subacute	Inhibitory 1 Hz, contralesional M1, 10 sessions	Parallel-group (sham vs. rTMS), double-blind, randomized	BBT	Subcortical vs. cortical involvement
Ludemann-Podubecka et al. (20)	40	Subacute	Inhibitory 1 Hz, contralesional M1, 15 sessions	Parallel-group (sham vs. rTMS), double-blind, randomized	WMFT, MESUPES, index finger tapping, cortical excitability (MEP)	Lesion in dominant vs. non-dominant hemisphere
Chang et al. (21)	62	Subacute	Excitatory 10 Hz, ipsilesional M1, 10 sessions	Single-arm	FMA	Val/Val *BDNF* genotype, MEP response at BL
Lee et al. (22)	29	Subacute	Excitatory 10 Hz, ipsilesional M1, 10 sessions	Single-arm	FIM, K-MBI	Subcortical vs. cortical involvement, aphasia, mental status
Demirtas-Tatlidede et al. (23)	10	Chronic	Inhibitory 1 Hz, contralesional M1, 10 sessions	Single-arm	FMA, WMFT, mAS, hand grip strength	Integrity of transcallosal fibers
Ueda et al. (24)	25	Chronic	Inhibitory 1 Hz, contralesional M1, 12 sessions	Single-arm	WMFT	Cortical thickness
Ueda et al. (25)	30	Chronic	Inhibitory 1 Hz, contralesional M1, 10 sessions	Single-arm	FMA, WMFT, BRS	Laterality index in motor area
Ueda et al. (26)	25	Chronic	Inhibitory 1 Hz, contralesional M1, 12 sessions	Single-arm	FMA, WMFT	Integrity of CST
Hamaguchi et al. (27)	1,254	Chronic	Inhibitory 1 Hz, contralesional M1, 15 sessions	Single-arm, retrospective analysis	FMA	BL residual hand function
Tamashiro et al. (28)	59	Chronic	Inhibitory 1 Hz, contralesional M1, 21 sessions	Single-arm	FMA, WMFT, mAS	Hemispheric dominance
Kakuda et al. (29)	52	Chronic	Inhibitory 1 Hz, contralesional M1, 22 sessions	Single-arm, retrospective analysis	FMA, WMFT	BL residual hand function
Kakuda et al. (30)	204	Chronic	Inhibitory 1 Hz, contralesional M1, 22 sessions	Single-arm	FMA, WMFT	No effect of stroke subtype
Tatsuno et al. (31)	1,716	Chronic	Inhibitory 1 Hz, contralesional M1, 30 sessions	Single-arm, retrospective analysis	FMA	No effect of BL stroke severity
Carey et al. (32)	12	Chronic	Inhibitory 1 Hz with intermittent 6 Hz priming, contralesional M1, 5 sessions	Single-arm	Performance time in single hand component of TEMPA	PLIC volume, Beck Depression Inventory score
Brodie et al. (33)	22	Chronic	Excitatory 5 Hz, ipsilesional S1, single session	Parallel-group (sham vs. rTMS), single-blind, pseudo-randomized	Response time of goal-directed visuo-motor serial targeting task	White matter volume of ipsilesional S1
Uhm et al. (34)	22	Chronic	Excitatory 10 Hz, ipsilesional M1, single session	Crossover (sham vs. subthreshold rTMS vs. suprathreshold rTMS), rater-blinded, randomized	Cortical excitability (MEP)	Val/Val *BDNF* genotype
Kindred et al. (35)	14	Chronic	Excitatory 10 Hz, ipsilesional M1 AND inhibitory 1 Hz, contralesional M1, 3 sessions	Crossover (sham vs. inhibitory rTMS vs. excitatory rTMS), double-blind, randomized	Cortical excitability (RMT, MEP), walking speed	Structural connectivity of CST via tractography
Yozbatiran et al. (36)	12	Chronic	Excitatory 20 Hz, ipsilesional M1, single session	Single-arm	FMA, Barthel Index, ARAT, hand grip strength, 9-hole peg test, motion range of index finger and wrist	Age
Diekhoff-Krebs et al. (37)	14	Chronic	iTBS, ipsilesional M1, single session	Crossover (sham vs. rTMS)	JTT, index finger tapping, hand grip strength	Extent of CST damage, inhibition level from ipsilesional M1, excitation level from ipsilesional SMA
Lai et al. (38)	72	Chronic	iTBS, ipsilesional M1, 10 sessions	Parallel-group (sham vs. rTMS), double-blind, randomized	WFMT, Functional Ability Scale, reaction time task, index finger tapping	BL residual hand function
Ameli et al. (39)	29	Subacute + chronic	Excitatory 10 Hz, ipsilesional M1, single session	Crossover (sham vs. rTMS)	Index finger tapping & hand tapping	Subcortical vs. cortical involvement, lesion extension, fMRI activity of lesioned region
Emara et al. (40)	60	Subacute + chronic	Excitatory 5 Hz, ipsilesional M1 OR inhibitory 1 Hz, contralesional M1, 10 sessions	Parallel-group (sham vs. contralesional rTMS vs. ipsilesional rTMS), randomized	Activity Index	Subcortical vs. cortical involvement, total anterior circulation stroke
Niimi et al. (41)	62	Subacute + chronic	Inhibitory 1 Hz, contralesional M1, 22 sessions	Parallel-group (sham vs. rTMS), non-randomized	FMA, WMFT	pro*BDNF* level at BL

*rTMS, repetitive transcranial magnetic stimulation; iTBS, intermittent theta-burst stimulation; M1, primary motor cortex; S1, primary somatosensory cortex; aIPS, anterior intraparietal sulcus; dPMC, dorsal premotor cortex; PLIC, posterior limb of the internal capsule; SMA, supplementary motor area; CST, corticospinal tract; BDNF, brain-derived neurotrophic factor; DTI, diffusion tensor imaging; fMRI, functional magnetic resonance imaging; MEP, motor-evoked potential; RMT, resting motor threshold; AMT, active motor threshold; FMA, Fugl-Meyer Assessment; BBT, Box and Block Test; WMFT, Wolf Motor Function Test; MESUPES, Motor Evaluation Scale for Upper Extremity in Stroke Patients; FIM, Functional Independence Measure; K-MBI, Korean version of the modified Barthel Index; mAS, modified Ashworth Scale; BRS, Brunnstrom Recovery Stage; TEMPA, upper extremity performance test for elderly; ARAT, Action Research Arm Test; JTT, Jebsen Taylor Hand Function Test; BL, baseline*.

In terms of rTMS protocols, 10 studies used excitatory rTMS (17, 21, 22, 33, 34, 36, 39) or iTBS (18, 37, 38) targeting ipsilesional M1 (17, 18, 21, 22, 34, 36–39) or ipsilesional S1 (33), with either a single session (18, 33, 34, 36, 37, 39) or a total of 10 sessions (17, 21, 22, 38) of intervention. A total of 13 studies used inhibitory rTMS over contralesional M1, with a range from 5 up to 30 intervention sessions (19, 20, 23–32, 41). Two studies used both inhibitory contralesional, as well as excitatory ipsilesional rTMS over M1 (35, 40), and a single study measured the effects of a single session of excitatory contralesional rTMS on M1, dorsal premotor cortex (dPMC), and anterior intraparietal sulcus (aIPS) (16). rTMS/iTBS was either used as a stand-alone intervention, paired with conventional physiotherapy/occupational therapy respecting the core standards of practice or task-specific training of the affected limb (e.g., index finger tapping).

Clinical assessment of motor outcome varied greatly, with different studies using the Fugl-Meyer Assessment (FMA), Wolf Motor Function Test (WMFT), Box and Block Test (BBT), Barthel Index, index finger tapping frequency, maximal grip force, reaction time tasks, and others. The timing of the motor assessment also varied, with eight studies examining immediate effects after a single rTMS session (16, 18, 32–35, 37, 39), 13 studies examining motor improvement after a prescribed series of sessions (17, 19, 21, 24–31, 38, 41), and 5 studies examining improvement from 1 week to 6 months after completion of rTMS treatment (20, 22, 23, 36, 40).

For the purpose of this review, we defined acute stroke as < 2 weeks since stroke onset, chronic stroke as > 6 months since stroke onset, and subacute stroke as falling in between these two categories (12). Over half of the studies (16/26) included chronic patients only (23–38), three studies included subacute, as well as chronic patients (39–41), four studies included subacute patients only (19–22), and three studies included acute patients (16–18). The female to male ratio of all patients included in the studies was approximately 1:2 (males *n* = 2,596, 65.31%), with one study not reporting the sex of participants (40).

### Clinical Factors

#### Demographics

The majority of included studies described no association between certain demographic variables, such as age and sex, and treatment response (21, 22, 30–33). A single study of 12 chronic stroke patients found that increasing age was correlated with less recovery of motor function, as reflected by less increase in grip strength 1 h after a single session of excitatory rTMS over ipsilesional M1 (36).

#### Time Post-stroke

rTMS was shown to promote increased motor function when administered in the acute (<2 weeks), subacute (2 weeks−6 months), and chronic (>6 months) phase. Only a few studies directly examined time since stroke as a covariate for rTMS response and no association was found between time post-stroke and motor recovery after rTMS intervention (22, 30–32, 38). However, acute and subacute stroke patients were underrepresented, with 73% of studies investigating chronic patients.

#### Baseline Motor Impairment

Stroke patients with better motor function at baseline were more likely to respond to rTMS in several studies applying different rTMS protocols (29, 32). In a study by Emara et al. (40) subjects with better baseline motor function showed functional improvement, assessed by the Activity Index, after inhibitory 1 Hz rTMS over contralesional M1. In contrast, patients with worse baseline function showed no improvement (40). However, in the same study, when applying excitatory 5 Hz rTMS over ipsilesional M1 in a different cohort, these patients showed significant functional improvement following the intervention regardless of their baseline motor function (40). Recent clinical guideline recommendations indicate a superior efficacy of inhibitory contralesional rTMS over excitatory ipsilesional rTMS (12). However, in the study of Emara et al. (40) more heavily affected patients responded to excitatory ipsilesional rTMS only, which leads to this rTMS protocol appearing more efficient.

Similarly, Hamaguchi et al. (27) retrospectively investigated 1,254 stroke patients who received inhibitory 1 Hz rTMS over contralesional M1. They reported that stroke patients with severe and moderate initial motor impairment were more likely than patients with mild initial motor impairment to show improvement in the FMA after an rTMS intervention (27). The authors suggested that functional improvement resulting from rTMS based treatment is in general lower in patients with better baseline function. However, in a more recent study, the same research group retrospectively analyzed 1,716 stroke patients receiving the same rTMS protocol (inhibitory 1 Hz rTMS over contralesional M1) and reported that the level of initial motor impairment was not significantly associated with rTMS response (31). Patients with low to high levels of baseline motor capacity showed significant motor improvement in the FMA after rTMS intervention, and no significant difference was found between patients with low or high baseline functioning (31).

### Structural Imaging Factors

#### Subcortical vs. Cortical Lesion Location

The beneficial effect of rTMS for promoting motor recovery in stroke patients has been suggested to be associated with the specific lesion location (19, 39, 40). In general, stroke patients with purely subcortical lesions and a spared cortex tend to show greater beneficial effects of rTMS than stroke patients with additional cortical involvement (19, 39, 40).

In a recent study by Kim et al. (19) 10 sessions of inhibitory (1 Hz) rTMS over contralesional M1 had a beneficial effect on upper limb motor recovery in the BBT in stroke patients with purely subcortical lesions. A significant improvement on the Brunnstrom stage of the affected hand immediately after rTMS intervention and at 1-month of follow-up were reported in the subgroup of subcortical stroke patients. In patients with additional cortical involvement, no beneficial effects of rTMS were seen (19).

In another study using a high-frequency rTMS protocol of 10 Hz over ipsilesional M1, beneficial rTMS-effects on frequency and amplitude of index finger tapping and hand tapping of the affected hand were seen in 14 of 16 purely subcortical stroke patients, while recovery was only seen in 7 of 13 patients with additional cortical involvement (39). In fact, a slight, non-significant dexterity deterioration of the affected hand was seen after rTMS intervention in the latter group (39).

Emara et al. (40) applied excitatory rTMS (5 Hz) over ipsilesional M1 and reported a beneficial rTMS effect on functional recovery, measured via the Activity Index, in subcortical, as well as cortical stroke patients. In the same study, a different set of patients received inhibitory 1 Hz rTMS over contralesional M1 and only patients with purely subcortical lesions showed a beneficial rTMS effect. Patients with cortical involvement did not respond to rTMS treatment. In this study, the authors distinguished between total anterior circulation stroke, partial anterior circulation stroke, posterior circulation stroke and lacunar stroke, rather than specifying the exact location of the lesion. Patients were categorized based on the presence or absence of cortical lesion involvement as assessed via MRI. The authors proposed that for the restoration of interhemispheric balance through contralesional rTMS, an intact ipsilesional cortex is a prerequisite, which is not given in stroke patients with cortical involvement (40). However, this proposition has to be considered carefully, as it is questionable whether the ipsilesional cortex of a patient with a stroke in a non-cortical area can truly be considered “intact,” due to the effects of diaschisis (43).

All three studies examining the effect of lesion location on rTMS response have shown that rTMS seems to have a more beneficial effect in subcortical stroke patients than in patients with additional cortical involvement. However, none of the three studies adjusted their statistical analyses for lesion size. Future studies need to control for lesion size to rule out that the more beneficial rTMS effect in subcortical patients is not driven by an overall smaller lesion size in those patients compared to patients with cortical lesion extension.

#### Lesion Extension, Gray Matter, and White Matter

Several structural brain properties, such as lesion extension, cortical thickness, and white matter (WM) characteristics have been proposed to be associated with the degree of rTMS response in stroke patients with motor deficits (24, 32, 33, 39, 40).

A negative association between lesion extension and rTMS response was reported in a study applying excitatory rTMS over ipsilesional M1 (39). In that study, a larger lesion extension with involvement of cortical motor areas was related to poorer motor improvement after rTMS intervention, as measured via index finger and hand tapping. The influence of lesion extension on rTMS response was supported in another study reporting that patients with total anterior circulation stroke (i.e., a cortical stroke affecting brain areas supplied by the anterior branches of the middle as well as anterior cerebral artery resulting in a large lesion volume) showed a significantly lower level of motor recovery, measured using the Activity Index, after rTMS compared to patients with partial posterior middle cerebral artery stroke, posterior circulation stroke, and lacunar stroke (40).

Further, a positive correlation between cortical thickness of the postcentral and supramarginal gyrus of the affected hemisphere and improved motor recovery, assessed using the WMFT, after rTMS intervention was reported in a recent study applying inhibitory 1 Hz rTMS over contralesional M1 (24). The lesioned hemisphere showed a significant thinner cortical thickness compared to the unaffected hemisphere. However, this association was only reported for cortical thickness but not for the overall GM volume.

After excitatory rTMS over the ipsilesional primary somatosensory cortex (S1), a positive correlation was observed between residual WM volume of the ipsilesional S1 and motor improvement of a goal-directed visuo-motor serial targeting task (33). No association was found between the degree of motor improvement and residual WM volume of ipsilesional M1, residual GM volume of ipsilesional S1 and residual GM volume of ipsilesional M1. In a combined linear regression model including age and GM volume, as well as WM volume of ipsilesional S1, 72% of the variance could be explained (*p* = 0.042), while no statistical significance was reached when excluding WM volume of ipsilesional S1 (*r*^2^ = 0.244, *p* = 0.376) (33).

The association between WM preservation and motor recovery after rTMS was further investigated in a study using low-frequency (1 Hz) rTMS over contralesional M1. Each 1 Hz rTMS session was preceded by a session of 10 min of intermittent 6 Hz rTMS over the same motor hotspot (32). Priming the motor hotspot with 6 Hz stimulation was previously reported to accentuate the effects of low-frequency rTMS in healthy subjects (44). The authors found a positive association between the preserved volume of the ipsilesional posterior limb of the internal capsule (PLIC) and the level of rTMS response, as measured by performance time in a single-hand component of the TEMPA performance test. The preservation of other ipsilesional motor network regions [i.e., M1, S1, premotor cortex (PMC) and supplementary motor area (SMA)] was not associated with rTMS response (32). However, this study from Carey et al. (32) is the only one included in this review performing a priming prior to the rTMS session. Primed rTMS is a rarely used approach in the rTMS literature and its benefits over unprimed rTMS remain unclear.

### Connectivity and Functional Imaging Factors

#### Corticospinal and Transcallosal Tract Integrity

Although stroke is classically described as causing neurological deficits by affecting localized, specific brain areas (45), a growing body of research demonstrates the importance of network effects resulting from disruption of communication between distant brain regions (46–48). Techniques such as diffusion weighted imaging (DWI) or diffusion tensor imaging (DTI), correlations between functional magnetic resonance imaging (fMRI) signals in different regions, and assessment of the amplitude and latency of motor-evoked potentials (MEPs) from TMS can be used to assess the structural and functional connectivity of different cortical and subcortical structures (48). Several studies applying those techniques have found that the integrity of corticospinal and transcallosal tracts are specifically associated with response to rTMS (16, 23, 26, 35, 37).

Corticospinal tract (CST) integrity appears to be a particularly important clinical marker for the ability to respond to rTMS intervention. In a study of chronic stroke patients, Kindred et al. (35) showed that higher baseline structural connectivity between M1 and the CST, measured as the sum of streamlines assessed by DTI, was positively associated with a greater decrease in MEP latency after a single session of inhibitory or excitatory rTMS. Similarly, Ueda et al. (26) demonstrated that certain DTI measures of CST integrity (mean and radial diffusivity) showed a positive correlation with motor improvement on the FMA and WMFT after 12 sessions of inhibitory 1 Hz rTMS over the contralesional M1, although no correlations were seen with other key measures, such as fractional anisotropy. A study using a single session of excitatory iTBS over ipsilesional M1 showed a significant negative correlation between the degree of CST damage and improvement of motor function in multiple measures (i.e., Jebsen-Taylor Hand Function Test [JTT], index finger tapping, hand grip) (37). CST damage was estimated based on the individual lesion intersection volume, relative to the total CST volume.

Baseline presence and strength of MEPs following TMS stimulation of the motor cortex, an electrophysiological measure that depends on CST integrity, has also been shown to be significantly associated with improvement of motor function after both excitatory iTBS and excitatory 10 Hz rTMS (21, 22, 38). Specifically, Chang et al. (21) found that patients who had an MEP response at baseline were 2.14 times as likely to have clinically meaningful motor improvement as assessed with the FMA (*p* = 0.044) after 10 Hz rTMS over ipsilesional M1 than patients with no MEP response. They did, however, not find an equivalent correlation between motor improvement and DTI measures of CST integrity (21), suggesting that physiological measures using single pulse TMS may be more sensitive than current anatomical imaging measures.

A positive linear relationship was reported between motor improvement (assessed with the FMA) after inhibitory 1 Hz rTMS over contralesional M1, and transcallosal fiber integrity between contralesional M1 and ipsilesional M1 (23). Higher fractional anisotropy values were associated with better motor recovery, highlighting the crucial role of interhemispheric communication for neural reorganization and motor recovery after stroke.

Diekhoff-Krebs et al. (37) combined several fMRI, TMS and clinical assessment parameters in a multivariate prediction model to assess which parameters allow the best prediction of motor improvement after a single session of excitatory iTBS over ipsilesional M1. They came to the conclusion that dynamic causal modeling (DCM) of endogenous connectivity parameters in a motor network, consisting of bihemispheric M1, PMC and SMA, and the clinical deficits assessed prior to stimulation with ARAT, allowed the best prediction of motor improvement after iTBS, explaining 82% of the variance (*p* = 0.016) (37). Those results indicate that brain connectivity parameters and initial motor function are stronger predictors for individual's motor recovery after iTBS than any TMS parameters, which did not further improve the prediction model (37). However, this model has yet to be validated as a predictor of rTMS response in studies evaluating long-term recovery.

#### Other Functional Imaging Factors

Baseline activity of motor areas, individual connectivity patterns within the sensorimotor network prior to rTMS treatment, and hemispheric dominance have been associated with motor improvement after rTMS and can be measured via fMRI and functional near-infrared spectroscopy (fNIRS) (20, 25, 28, 37, 39).

Initial activity of ipsilesional M1 prior to rTMS intervention was positively correlated with motor improvement of index finger tapping after excitatory rTMS over that area (39). At baseline, patients that responded positively to rTMS showed widespread blood oxygen level dependent (BOLD) activation in the ipsilesional, as well as contralesional hemisphere during movements of the affected hand in a task-based fMRI design. The BOLD signal is a ratio between oxygenated and deoxygenated hemoglobin and is therefore a measure of neuronal metabolism that is highly correlated with and often used synonymously with neuronal activation (49). In contrast, patients that did not respond to rTMS showed weaker neural activity in both hemispheres, especially in ipsilesional M1, during hand movement (39). In another study, Diekhoff-Krebs et al. (37) reported that both stronger excitatory coupling between ipsilesional M1 and ipsilesional SMA and stronger inhibitory effects of ipsilesional M1 on contralesional M1 at baseline were associated with better motor recovery (measured using JTT, hand grip, and index finger tapping) following iTBS.

The individual activity of S1, PMC, and SMA prior to treatment was also associated with motor outcomes after rTMS. Patients with dominant neural activity of those motor areas of the unaffected hemisphere had significantly better motor recovery in the FMA, WMFT, and modified Ashworth Scale after inhibitory contralesional rTMS compared to patients with a dominant motor network activity in the lesioned hemisphere (28). Hemispheric dominance was assessed by calculating laterality indices based on changes in oxy-hemoglobin during a motor task-based fNIRS assessment. Consistent with previous fNIRS findings, a more recent fMRI study supports the more beneficial effect of inhibitory rTMS over contralesional M1 on the FMA, WMFT, and Brunnstrom stage in patients with dominant motor network activity in the unaffected hemisphere, in contrast to patients with dominant motor network activity in the lesioned hemisphere (25).

A stroke lesion in the hemisphere, representing the dominant hand of the subject, was associated with poor motor recovery in the sham condition as assessed by the WMFT, Motor Evaluation Scale for Upper Extremity in Stroke Patients (MESUPES), index finger tapping, and MEPs, but showed better motor recovery after rTMS, as reported in a study using inhibitory rTMS over contralesional M1 (20). In contrast, patients with a lesion in the hemisphere representing the non-dominant hand, showed equal motor recovery in the sham and the rTMS condition.

### Genetic Factors

Brain-derived neurotrophic factor (*BDNF*) is a neurotrophic protein involved in a variety of key neurological processes, including memory consolidation and neuroplasticity (50). A relatively common single-nucleotide polymorphism (Val66Met) in the *BDNF* gene has been associated with decreased neuroplasticity in response to TMS and TBS. This *BDNF* gene polymorphism is thus a promising candidate for a negative clinical predictive factor (51). Indeed, MEP amplitudes in response to 10 Hz rTMS over ipsilesional M1 were higher in stroke patients homozygous for the Val-allele in contrast to heterozygous patients or patients homozygous for the Met-allele (34).

In a study of clinical motor improvement, Chang et al. (17) reported significantly greater improvement in upper limb motor function (FMA and BBT) at up to 2 months in Val/Val patients following 10 sessions of 10 Hz rTMS over ipsilesional M1 vs. those with at least one Met allele. Similarly, they reported in a separate study that patients with the Val/Val genotype were 1.8 times more likely (*p* = 0.016, odds ratio: 6.05, 95% confidence interval: 1.39–26.27) to show improved motor function using the FMA following 10 sessions of 10 Hz rTMS over ipsilesional M1 (21). Along the same lines, patients with low baseline serum levels of pro-*BDNF*, the precursor form of *BDNF*, were significantly more likely to respond to 1 Hz rTMS over contralesional M1 compared to patients with high levels of pro-*BDNF*, as measured by the FMA and WMFT. This was possibly due to having a greater proportion of activated *BDNF* (41).

However, Niimi et al. (41) found no effect of *BDNF* genotype on response to rTMS treatment in the FMA and WMFT in a study of contralesional 1 Hz rTMS. It thus appears that the efficacy of ipsilesional rTMS protocols is affected more by the Val66Met polymorphism.

### Miscellaneous Factors

Several included studies investigated the effect of baseline functional status and comorbidities, showing negative correlations between rTMS efficacy in promoting motor recovery and the presence of aphasia, a lower Mini-Mental State Examination (MMSE) score (indicating lower function), and a higher score on the Beck Depression Inventory (indicating greater severity of comorbid depression) (22, 32).

## Discussion

**Figure 4:**
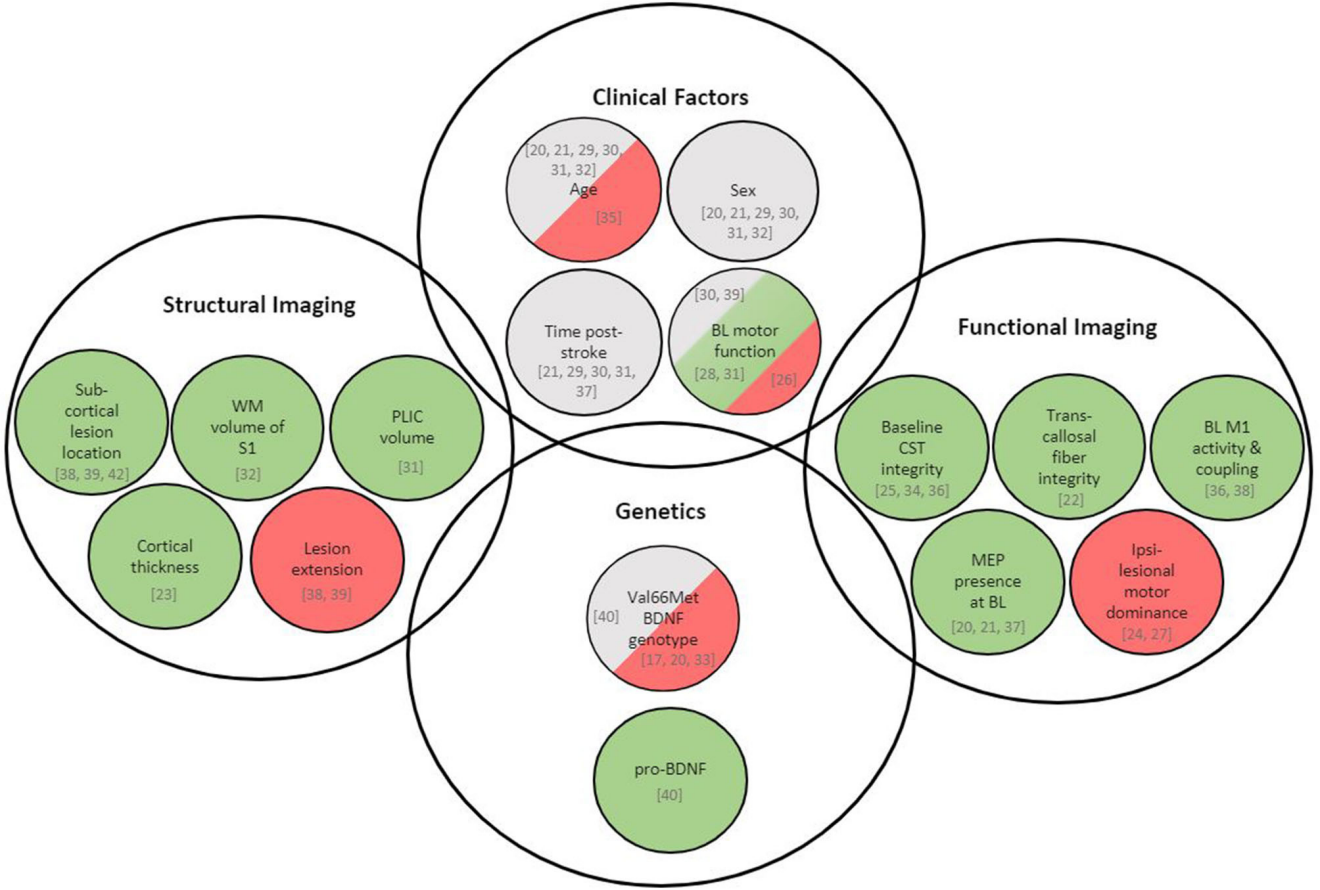
Overview of variables associated with rTMS-induced recovery in stroke patients. Green represents variables being positively associated with rTMS response, red represents variables being negatively associated with rTMS response, and gray represents variables showing no association with rTMS response. Respective literature is specified in square brackets. *BDNF*, brain-derived neurotrophic factor; BOLD, blood oxygen level dependent; CST, corticospinal tract; MEP, motor-evoked potential; M1, primary motor cortex; PLIC, posterior limb of internal capsule; S1, primary somatosensory cortex; WM, white matter; BL, baseline.

The purpose of this review was to identify clinical, structural, and neurobiological factors in human subjects that may be associated with greater response to rTMS for stroke rehabilitation. Factors most consistently associated with rTMS response were biomarkers of structural and functional integrity of motor networks whereas the role of clinical, demographic and genetic factors is less certain (Figure 4). We discuss trends and conclusions we can draw from the current literature and how we can move further the state of knowledge, specifically how the identification of potential predictors could be accelerated by efficiently combining pre-clinical and clinical research efforts.

### Predictive Factors for rTMS Response

#### Clinical and Demographic Factors

It is well known that older age (≥65 years) and female sex increase the probability of severe deficits and poorer functional outcomes in stroke patients in general (52, 53). Whether and how these risk factors translate into a differential response to specific rehabilitation interventions in general and specifically to brain stimulation interventions remains unclear. The studies reviewed here report heterogeneous effects of age and no effect of sex on the response to TMS interventions. One study reports a positive association between increasing age and less recovery of motor function after rTMS (36). However, increasing age is generally associated with worse baseline motor impairment after stroke (54). Therefore, it needs to be further investigated whether the reported negative association between age and rTMS response remains significant after adjusting for baseline motor impairment. None of the reported studies were specifically designed or sufficiently powered to investigate the role of age and sex on rTMS response. Specifically designing future studies to directly examine potential effects of sex and age on rTMS response might reveal associations that may have been missed when including those parameters as covariates only.

Not only the effect of age and sex, but also the impact of baseline motor impairment remains uncertain based on the reviewed studies. While most studies performing contralesional inhibitory rTMS report better improvements in mild to moderately affected patients (29, 32, 40), such a relationship has not been established for other protocols. Two studies report no effect of baseline impairment or even report better improvement in more severely affected patients (27, 31). The impact of baseline motor impairments on the potential efficacy of rTMS treatment for stroke recovery is thus equivocal and it remains unclear if results may be related to evaluation tools sensitivity and ceiling effects.

Pre-clinical studies have provided clear evidence linking cortical lesion volume and location, behavioral recovery and reorganization in distant spared brain areas (55–57). After small lesions in the motor cortex that induce mild deficits, there is a decrease of cortical territory from which hand movements can be elicited in both the ipsi- and contralesional hemisphere. In contrast, more impaired animals with bigger lesions show larger motor representations in the same areas. Thus, the role of spared motor areas, such as the contralesional M1, and consequently the effect on recovery of rTMS treatment targeting these motor areas, are very likely to vary based on lesion characteristics and impairments. In fact, when considering the impact of these factors on the physiological reorganization of spared motor areas in animal studies, one could predict that a given treatment (i.e., 1 Hz inhibitory protocol over the contralesional M1) should have opposite effects in mildly and severely affected patients. It has been shown for example that excitatory stimulation of the contralesional cortex in rats with corticospinal tract lesions favors anatomical rewiring and behavioral recovery (58). Excitatory stimulation of the contralesional hemisphere may thus be the preferred approach in cases where lesions largely or completely disconnect the ipsilesional hemisphere from the contralateral spinal cord.

#### Integrity of Motor Network

Several aspects of structural and functional motor network integrity appeared throughout the reviewed studies as areas in which further research may be able to identify robust predictive factors (Figure 4). Specifically, a relatively preserved ipsilesional M1 with its intra- and interhemispheric connectivity seems to be related to a favorable rTMS response. Stroke can lead to a disruption of neural signal transmission due to changes in axon diameter and changes in the myelination of white matter tracts (39). It has been hypothesized that this disruption of structural and functional connectivity may hinder the propagation of rTMS-modulated cortical activity from the site of stimulation throughout the motor network (39, 42, 59). A certain degree of preserved descending white matter projections, as well as functional motor network connectivity, may thus be needed for rTMS-induced changes in neural activity to manifest into improved motor behavior (39). The importance of white-matter integrity for recovery is also supported by pre-clinical data. In monkeys, the extent of recovery of hand and digit function correlates to both white and gray matter volume damage (60). However, recovery is slower after brain injuries that include frontal white matter in comparison to lesions of similar or even greater volumes, but restricted to gray matter (60). For the assessment of structural network integrity with respect to rTMS response, white matter markers thus appear to be more important than markers of gray matter. Not surprisingly, large lesions and the presence of extensive cortical damage limit the effect of TMS (as would be the case with any rehabilitation intervention). However, for less extensive or mainly subcortical lesions, measures of WM integrity such as WM volume of the CST, the internal capsule or transcallosal fibers seem to be better markers.

Transcallosal fiber integrity between contralesional M1 and ipsilesional M1 has a positive linear relationship with motor improvement, assessed via the FMA, after inhibitory 1 Hz rTMS over contralesional M1 (23). Fractional anisotropy (FA), a diffusion tensor imaging-based parameter reflecting the orientation of white matter fiber bundles by measuring water diffusivity, was used to examine microstructural damage of transcallosal motor fibers between ipsilesional and contralesional M1. Higher FA values were associated with better motor improvement after rTMS (23), reflecting a potential predictive role of the integrity of the corpus callosum in rTMS response. Several other studies have reported lower corpus callosum FA to be associated with poorer motor outcomes (61–65), highlighting the important role of transcallosal fiber integrity in motor recovery and interhemispheric reorganization post-stroke.

Future studies specifically targeting the predictive role of transcallosal fiber integrity in rTMS-elicited motor improvement are necessary. A systematic review from Bertolucci et al. (66) specifically looked at interhemispheric effects after stroke, assessed with TMS, and the relationship with motor recovery. The authors suggest that the modulation of transcallosal inhibition could be of benefit for stroke patients with good residual motor function and strong interhemispheric inhibition, but less for patients with poor residual motor function and weak interhemispheric inhibition (66). For assessment of transcallosal fiber integrity, two electrophysiological TMS approaches have been used: (a) the ipsilateral silent period (iSP) of single TMS pulses, in which a longer iSP duration and a higher iSP magnitude represent more transcallosal fiber damage, and (b) the TMS double pulse paradigm (66). The measurement of TMS-induced electrophysiological response with a combined TMS-EEG technique provides an alternative approach for assessing interhemispheric inhibition and transcallosal fiber damage (67).

Several of the identified studies in this review showed that patients with purely subcortical stroke were more likely to have a greater response to rTMS intervention than patients with cortical involvement. In cortical stroke, intracortical inhibition is suppressed (42, 68). Reduced inhibition in the ipsilesional hemisphere drives a downregulation of inhibitory activity in the contralesional hemisphere through axonal connections (69). The loss of intracortical inhibition is associated with enhanced excitatory activity in the immediate neighborhood of the cortical lesion (68). Those changes in inhibitory as well as excitatory mechanisms in the cortex might play a role in the inferior rTMS response in patients with cortical stroke compared to patients with subcortical stroke (19, 39). However, it needs to be taken into account that some rTMS studies report no significant association between lesion location and motor improvement after rTMS intervention in stroke patients (21, 30, 38).

These findings highlight that our current understanding of the effects of lesion location and volume on rTMS treatment efficacy is still quite limited. These interactions are likely to be very complex given the heterogeneity of lesion characteristics across patients. They might therefore benefit from pre-clinical studies that can better isolate and dissect the variables involved. As stated above, lesion location and volume affect the physiological reorganization in distant areas, within the ipsi- and contralesional motor network. Thus, perhaps the most reliable biomarkers of the effectiveness of rTMS approaches will be based on assessment of the functional state of spared areas in the motor network.

Only a few studies examined the predictive role of functional imaging on rTMS response. An association between stronger overall BOLD activation at baseline (39), as well as stronger intrahemispheric excitatory coupling between motor areas (37) and rTMS response, have been reported in two studies using fMRI. An association between dominant neuronal activity in the unaffected in contrast to the lesioned hemisphere and rTMS response has been shown in an fNIRS study (28) and was confirmed in another fMRI study (25). The number of studies using functional neuroimaging techniques such as fMRI and fNIRS for rTMS response prediction is limited and more studies are needed in order to draw solid conclusions from these findings.

Three studies reported that stroke patients with the presence of motor-evoked potentials (MEPs) of the paretic *first dorsal interosseous* muscle (FDI) (21, 38) or the *abductor pollicis brevis* muscle (APB) (22) after single-pulse TMS at baseline are more likely to have clinically meaningful motor improvement after rTMS intervention than patients with no initial MEP response. The presence or absence of MEPs informs about the functional integrity as well as cortical excitability of the motor network and the cerebrospinal tract (21, 22, 38).

#### *BDNF* and Synaptic Plasticity

The only genetic factor for rTMS response investigated was the presence of the Val66Met *BDNF* polymorphism, which was shown in several studies to be negatively associated with motor response to excitatory ipsilesional rTMS (17, 21, 34). Given the known role of *BDNF* in brain plasticity (50) and the theorized importance of modifying synaptic connections in the rTMS-aided recovery process from stroke (70), it is reasonable that a loss-of-function mutation in this gene would be associated with a smaller response to the treatment. This concept is strengthened by the evidence that a greater proportion of activated *BDNF* in the circulation seems to be associated with greater response to rTMS (17, 21).

This association between *BDNF* genotype and rTMS response has however not been confirmed in a separate study utilizing inhibitory contralesional rTMS (41). Some evidence from pre-clinical studies in rats seems to suggest that non-invasive brain stimulation may directly increase the expression of *BDNF*, facilitate neurogenesis (71) and enhance *BDNF* affinity for tyrosine receptor kinase B (TrkB), a neurotrophin receptor (72). This would result in a direct modulation of synaptic plasticity mediated by *BDNF*-TrkB-NDMA receptors (73). Such mechanisms may be of greater relevance in the perilesional cortex, but much less so in the contralesional unaffected cortex. If so, they would provide a pathophysiological rational for a possible link between the stimulation protocol and the specific genotype effect. Further research will be needed to ascertain if this association between genotype and rTMS protocol is valid and TMS studies in animal models may be a more efficient way to reliably demonstrate this specific interaction than clinical trials.

#### Stand-Alone vs. Combined rTMS Approach

The majority of studies included in this review (*n* = 19/26) used some form behavioral intervention in addition to rTMS. Standardized physical or occupational therapy was used in 13 studies (19, 21, 24–31, 38, 40, 41) and 6 studies combined rTMS with task-specific training (e.g., index finger tapping practice, visuomotor serial targeting task) (16, 20, 32, 33, 35, 39). Only 7 studies used rTMS as stand-alone therapy without any additional behavioral intention (17, 18, 22, 23, 34, 36, 37). Up to date, no study directly investigated, if the value of potential predictive factors for rTMS response differs depending on whether rTMS is used in a stand-alone or a combined approach.

Basic neuroimaging studies on the effect of non-invasive brain stimulation on cerebral activity seem to suggest that a modulatory effect of non-invasive brain stimulation on cerebral activity is caused by the interaction of stimulation and physiological recruitment of cortical neuronal activity. In a previous neuroimaging study of the motor system, it has been shown that tDCS stimulation alone has no effect on cortical activity but inhibitory or excitatory modulation was demonstrated in healthy subjects while simultaneously performing a motor task (74). Similar physiological neuromodulatory effects have also been reported for the language system, where a change in cerebral blood flow as surrogate marker of cortical activity was only observed as interaction between rTMS and a verb generation task, but not when applying rTMS alone (75).

Based on those findings, it is reasonable to assume that the potential predictive factors reported in this review apply more to rTMS used in a combined approach than rTMS as a stand-alone intervention. Since the literature evidence for rTMS as stand-alone therapy is inconclusive, it has been recommended for future studies to combine rTMS with some form of standardized therapy given to sham as well as intervention groups to minimize variability arising from non-standardized forms and doses of therapy and clearly isolate the add-on effect of brain stimulation (12).

## Limitations of Existing Data

Only a few large, controlled studies that investigate predictive factors for rTMS response in stroke patients with motor deficits currently exist. More than half (14/26) of the included studies had a small sample size (*n* ≤ 30), and more than 60% (16/26) of studies had no sham control condition. Only three of the included studies were randomized controlled trials with an adequate control group. Difficulties with patient recruitment, feasibility, and financial resources make carrying out these studies difficult. The lack of large, controlled trials limits the conclusions that can be drawn from the data, especially with the heterogeneity of study design and large variability in methodology.

rTMS protocols used in the included studies varied regarding stimulation frequency and intensity, targeted brain region, stimulated hemisphere, and number of therapeutic sessions (ranging between 1 and 30). All these factors are known to affect the extent of cortical excitation, and presumably underlying molecular mechanisms (76–78). Besides heterogeneous study protocols and designs, the inclusion and exclusion criteria for patient selection also varied between studies. Some studies excluded patients with aphasia, cognitive impairment, comorbidities, or ongoing medication usage, while in others these patients were included. While pre-clinical studies examining these factors are sparse, they can have effects on rTMS protocol efficacy. For example, the cognitive state of aged rats prior to the rTMS protocol was shown to affect the impact of the treatment on behavioral performance (79).

Additionally, outcome measures and timing of assessment varied greatly between studies. While some studies measured motor activity immediately after a single rTMS session, others measured motor improvement after finishing the whole set of rTMS sessions. Five studies investigated long-term effects of rTMS by assessing the recovery status at 1 up to 4 follow-up appointments, between 1 week up to 6 months after rTMS treatment (20, 22, 23, 36, 40). However, time interval between rTMS intervention and follow-up session, motor outcome measures as well as statistical tests in data analyses strongly varied. All these factors can be considered as potential biases. Most studies used clinical assessment tools for evaluation of motor function. However, other studies used change in cortical excitability through resting motor threshold (RMT), MEP and active motor threshold (AMT) as surrogate for motor function. Clinical assessment of motor function included among others the FMA, WMFT, BBT, Barthel Index, index finger tapping frequency, maximal grip force, and reaction time tasks. However, it should be noted that the majority of these tests measure the degree of motor impairment. Only four studies (22, 36, 38, 40) used evaluations of restriction in activities and participation, which is often a more relevant measure for a patient's daily functioning (80). Future studies should use measures of both motor impairment and evaluations of restriction in activities and participation in daily life, in order to better quantify the benefit of rTMS intervention for patients. In particular, they should include standardized measures of sensorimotor recovery after stroke (81).

It should also be noted that 25/26 included studies examined upper limb motor function, while only a single study assessed gait and lower limb function (35). The lack of studies assessing the effect of rTMS on lower limb performance post-stroke needs to be addressed in future studies.

Finally, in some of the included studies, rTMS was used as the sole intervention (17, 18, 22, 23, 34, 36, 37) without concurrent physiotherapy, occupational therapy or specific task-based training. In animals, treatments that promote plasticity and recovery after central nervous system injuries are typically more effective when combined with rehabilitation (82). All of these widely differing design parameters across the studies make it difficult to directly compare findings and interpret the results as a unified whole.

This review includes two retrospective studies with a large stroke patient population of *n* = 1,254 (27) and *n* = 1,716 (31). However, both studies were conducted in the same research group and included patients from the same data pool, receiving inhibitory 1 Hz rTMS over contralesional M1. When investigating differences in potential predictive factors between different rTMS protocols, it has to be taken into account that the two above-mentioned studies account for nearly 75% of the total patient population included in this review, leading to an over-representation of the inhibitory contralesional rTMS protocol and the potential repetition of data.

Over half of the included studies in this review (14/26) were conducted by research groups in Japan and South Korea, demonstrating the leading contributions of these nations in rTMS research. As only studies in English, French, or German were considered for inclusion in this review, relevant studies published in other languages may have been missed.

Finally, it must be stated that it was not the purpose of this paper to provide a comprehensive and systematic review of all possible predictive factors, but rather to identify factors that may be good candidates to be further explored in targeted studies. As such the scope of the review was limited to only one database.

## Future Perspectives

The high variability in study design and rTMS parameters between studies reveals the importance of standardization and homogenization of rTMS trials in the future. Findings can only be compared properly if study design and rTMS parameters, such as stimulation intensity and frequency, number of sessions, targeted brain area and hemisphere, and outcome measures are consistent between studies. To ensure such standardization, the design of future rTMS trials in stroke patients with motor deficits should be informed by expert consensus such as the *CanStim consensus recommendations for rTMS in upper extremity motor stroke rehabilitation trials* (12) regarding patient population, rehabilitation interventions, outcome measures, and stimulation parameters.

Similar expert recommendations are available e.g., for the use of kinematic and kinetic movement quantification tools as well as qualitative measures of motor performance of the upper limb as developed by the Second *Stroke Recovery and Rehabilitation Roundtable* (83).

This review has revealed several knowledge gaps that should be addressed in future clinical trials. *BDNF* genotype has been shown to be associated with motor recovery after excitatory ipsilesional rTMS, but not after inhibitory contralesional rTMS. Future clinical trials need to address the differential response to these two procedures in patients with *BDNF* polymorphism and reveal potential associations between applied rTMS protocol and *BDNF* genotype.

As the majority of stroke patients included in this review were in the chronic phase (73%, Figure 3), more studies in acute and subacute stroke patients are needed to further investigate predictive factors for rTMS response on recovery in those earlier stages post-stroke, specifically since stroke rehabilitation in most health care systems is provided during these early phases. Moreover, in the majority of patients in this review, the effect of rTMS on recovery was assessed immediately after finishing all intervention sessions. Follow-up assessment of motor function was only performed across a few trials. Future trials should ensure that patients are monitored longitudinally to potentially identify associations between predictive clinical and imaging baseline factors and longitudinal motor outcomes.

Finally, perhaps the most disconcerting realization at this stage of implementation is the apparent lack of understanding of the mechanisms through which rTMS protocols can increase recovery after stroke. We believe that it is likely that pre-clinical research may be the most needed and useful to answer these questions. For example, it was recently shown that high-frequency rTMS can reduce apoptotic cell death and promote neuronal sprouting of cortical projections in mice after stroke (84). Using invasive methods, animal models can also inform about the effects of rTMS on the neural network activity and how these effects may vary following different types of strokes. These efforts will need to include models that reflect the complexity of the human sensorimotor cortical network (85, 86). Treatment parameters and selection criteria for human trials could thus be based on the directly measured effects of rTMS on the brain in suitable pre-clinical models rather than behavioral outcomes derived from clinical observations. In our view, such a systematic approach, could accelerate the translation process and make it more efficient because only such selection criteria are subject to clinical evaluation which are based on valid pathophysiological mechanisms with documented rTMS effects on the brain.

While it is unlikely that a single parameter will be sufficient to separate stroke patients likely to benefit from rTMS intervention from patients likely not to show rTMS response, the ultimate goal would be the development of a multivariate predictive model for rTMS response in stroke patients with motor deficits in order to optimize patient selection for specific rTMS interventions. By combining multiple predictive factors that may individually have low-to-moderate predictive ability, a more complete individual prediction model for rTMS response can be developed. The multivariate models developed by Diekhoff-Krebs et al. (37) for behavioral iTBS response combined endogenous connectivity parameters and clinical deficits at baseline and explained 82% of variance. Further development of such models, including other potential predictive factors identified in this review could enable a scoring system to be developed and validated for likelihood of response to rTMS, facilitating patient selection for clinical trial purposes.

However, it also needs to be considered that imaging techniques such as DWI/DTI, fMRI, and fNIRS are time-consuming and expensive procedures and thus difficult to implement in clinical settings. To be clinically useful, potential predictive factors should be easily determinable, preferably through routine structural imaging or blood lab tests.

A first attempt for a potential algorithm could be more general predictive scores for stroke recovery, such as the Predict Recovery Potential 2 (PREP2). This algorithm has a relatively good predictive accuracy (>70%), and can be calculated with clinical measures such as Shoulder Abduction and Finger Extension (SAFE) score and NIHHS score combined with TMS MEP measurements, thus allowing for calculation even when MRI or more complex imaging techniques are unavailable (87). Exploring the ability of modified versions of such algorithms to predict response to rTMS specifically may be another direction for further research.

## Conclusion

This review evaluated evidence for demographic, clinical, and neurobiological factors to distinguish stroke patients with motor deficits who are more likely to respond to rTMS intervention. Purely subcortical lesions, factors associated with an at least partially preserved ipsilesional motor network (undamaged M1, proper intra- and interhemispheric integrity of M1, well-preserved WM volume under the site of stimulation, and PLIC volume), as well as cortical thickness, motor network dominance in the unaffected hemisphere, and the absence of the Val66Met *BDNF* polymorphism are promising predictive factors. Based on the high variability in rTMS protocol and experimental design between studies, these findings need to be further investigated and confirmed in future research.

## Author Contributions

FH, JA, and AS contributed to the literature search. FH and AS contributed to drafting the manuscript, figures, and tables. A-UD-V and ND provided the portions of the manuscript regarding pre-clinical and animal data. AT, JE, and ND contributed to the concept of the review. AT supervised the project. All authors contributed to the revision and approval of the manuscript.

## Funding

This work was supported by the Canadian Partnership for Stroke Recovery and a Platform Support Grant of the Brain Canada Foundation. A-UD-V was supported by a postdoctoral fellowship from CONACYT, Mexico (CVU-330898).

## Conflict of Interest

The authors declare that the research was conducted in the absence of any commercial or financial relationships that could be construed as a potential conflict of interest.

## Publisher's Note

All claims expressed in this article are solely those of the authors and do not necessarily represent those of their affiliated organizations, or those of the publisher, the editors and the reviewers. Any product that may be evaluated in this article, or claim that may be made by its manufacturer, is not guaranteed or endorsed by the publisher.
